# Transcription and Activity of Digestive Enzymes of *Nezara viridula* Maintained on Different Plant Diets

**DOI:** 10.3389/fphys.2019.01553

**Published:** 2020-01-08

**Authors:** Pablo Emiliano Cantón, Bryony C. Bonning

**Affiliations:** Department of Entomology and Nematology, University of Florida, Gainesville, FL, United States

**Keywords:** diet, digestive enzymes, host plant, midgut, transcriptome, stink bug

## Abstract

*Nezara viridula* is a polyphagous stink bug that feeds on crops of economic importance such as corn, soybean and cotton. To increase understanding of the ability of this pest insect to feed on such diverse cropping systems, we analyzed the impact of an exclusive diet of corn or green bean on the enzymatic activity and transcriptomic profile of digestive enzymes. Growth rate and survival were reduced when insects were reared exclusively on green bean compared to corn. However, the overall protease and nuclease activity profiles were comparable between the two treatments. Distinct differences in inhibitor sensitivity and activity were seen in some cases, particularly for serine proteases in some regions of the midgut. The transcription profiles from *N. viridula* fed on corn versus green bean were distinct on principal component analysis of RNA-seq data. While specific transcripts differentially transcribed according to diet and across several tissues were identified, a large number of these transcripts remain unannotated. Further annotation for identification of these genes will be important for improved understanding of the remarkable polyphagy of *N. viridula*.

## Introduction

Crop damage resulting from insect herbivory is a primary source of economic loss in agriculture. Species that feed on multiple host plants are of particular concern, as they can move between adjacent crops, feeding on plants that mature at different times during the year or persist in refuge plants or grasses. Species of the family Pentatomidae (Hemiptera), such as the southern green stink bug, *Nezara viridula*, possess all of these traits (Panizzi, 1997). Changes in agricultural practices, such as reduction in insecticide use or no tillage strategies, as well as changes in climate, have allowed *N. viridula* along with other species in this family to rise in prominence as crop pests (Panizzi, 2015).

*Nezara viridula* is a cosmopolitan polyphagous species that feeds on the seeds and fruits of more than 100 plant species in 30 different families (Todd, 1989). Some of these plants include crops of high economic importance such as soybean, cotton, and corn (Tillman, 2010). *N. viridula* feeds by a piercing-sucking mechanism in two phases: digestion initiates with the extra-oral secretion of saliva with digestive enzymes such as trypsin and chymotrypsin into plant tissues. Heteropteran species such as *N. viridula* use a non-reflux system and a remarkable maneuverability of their stylets to pierce and deliver extra-oral secretions, which provides a rate of recovery of liquefied tissue of >90% (Cohen, 1995). Following ingestion, this plant matter is completely degraded and absorbed in the midgut (Lomate and Bonning, 2016). *N. viridula* has four anatomically and physiologically distinct midgut regions: M1, M2, M3, and M4 (Hirose et al., 2009). Most proteolytic activity occurs in M2 and M3 (Cantón and Bonning, 2019) mediated by the cysteine proteases Cathepsin B and L, while M4 functions to house endosymbiotic bacteria (Hosokawa et al., 2016). The proteases and nucleases present in saliva, salivary glands, and midgut tissues of *N. viridula* have been cataloged (Liu et al., 2018). The manner in which these myriad digestive enzymes are employed for digestion of plant material of varied composition is unclear.

The production of digestive protease inhibitors and a wealth of secondary compounds with toxic characteristics are among the main mechanisms by which plants defend against or deter herbivory. The regulation of insect response to plant defense mechanisms is of ongoing interest. In the case of protease inhibitors, insects can respond by general upregulation of digestive enzymes, production of specific enzymes that circumvent inhibition, or by detoxifying the toxic agents (Zhu-Salzman and Zeng, 2015). Some corn varieties produce cysteine proteases that damage the peritrophic membrane lining the insect gut (Pechan et al., 2000), but insects can upregulate inhibitors of these enzymes (Li et al., 2009). Changes in protease activities or gene transcription profiles have been noted in several insects including in response to phytotoxins (Halon et al., 2015), diet source (Coudron et al., 2007; Huang et al., 2017; Rivera-Vega et al., 2017), or adaptation to the presence of plant protease inhibitors (Lara et al., 2000; Oppert et al., 2005; Brioschi et al., 2007). Key proteases or defense mechanisms are potential targets for disruption for stink bug management through the application of protease inhibitors (Schlüter et al., 2010) or gene knockdown (Joga et al., 2016; Ghosh et al., 2017).

The goal of this study was to examine the effect of diet source on the proteases and nucleases of *N. viridula*. We used both transcriptomic and enzymatic assay approaches to generate data for gene transcription and biochemical profiles. Although enzyme activity profiles were similar, diet-dependent variation was sufficient to differentiate the transcriptomes derived from *N. viridula* maintained on corn versus green bean.

## Materials and Methods

### Reagents

For nuclease substrates, calf thymus DNA was obtained from Sigma-Aldrich (St. Louis, MO, United States) and baker’s yeast RNA was purchased from Fisher Scientific/Alfa Aesar (Haverhill, MA, United States). RNAlater stabilization solution was purchased from Invitrogen (Carlsbad, CA, United States). The protease substrates azocasein, N_α_ -Benzoyl-D,L-arginine 4-nitroanilide hydrochloride (BApNA), N-Succinyl-Ala-Ala-Pro-Phe p-nitroanilide (SAAPFpNA), L-Leucine p-nitroanilide (LpNA), pGlu-Pro-Leu p-nitroanilide (pGFLpNA) were obtained from Sigma-Aldrich. Z-Arg-Arg p-nitroanilide (zRRpNA) was acquired from Bachem (Bubendorf, Switzerland). The inhibitors Phenylmethylsulfonyl fluoride (PMSF), *N*_α_ -Tosyl-L-lysine chloromethyl ketone hydrochloride (TLCK), *N*_α_ -Tosyl-L-phenylalaninechloromethyl ketone (TPCK), E-64, Ethylenedintrilotetraecetic acid (EDTA) were also purchased from Sigma-Aldrich.

### Rearing and Dissection of *Nezara viridula*

The *N. viridula* colony was established August 12, 2014 with insects provided by Dr. Jeffrey Davis, Louisiana State University. The colony was reared on mixed diet at 28°C, 65% relative humidity, and a 16:8 hr light/dark photoperiod. The colony was supplemented once yearly with field caught *N. viridula* from Florida. For this study, *N. viridula* sub-colonies were maintained under the same conditions on exclusive diets of either corncobs with kernels (*Zea mays*) or organic green bean (*Phaseolus vulgaris*) pods. Diet was changed twice weekly. For containers with green beans, pods were arranged in a conical pile and complemented with a moist cotton plug in a 1 oz plastic cup. Cages were inspected each morning for the presence of adults. Following the molt to adult, insects were moved to a new container and allowed to feed for 24 h on their respective fresh diet prior to dissection the following morning. Salivary glands and midgut M1, M2, and M3 were dissected from adults in 0.1 M PBS, pH 7.4. For each biological replicate, tissues from approximately 12 adults were dissected and pooled for enzymatic assays. Midgut sections were dissected from five adults and salivary glands from 12 adults for RNA extraction. Tissues were either flash frozen in liquid nitrogen and stored at −80°C for protein extraction or submerged in RNAlater solution and kept at −20°C until RNA extraction was performed. Three biological replicates were conducted for all experiments.

### Assessment of Insect Growth Rate

For each diet, forty 2nd instar nymphs were put on corn or green bean diet and reared as described above. At each diet change, survival was recorded and the length from the end of the abdomen to tip of the head was measured for all live individuals. Adults were weighed. A two-tailed *t*-test was performed to evaluate significance in differences between the slope and standard error of the regression curves for insect growth (length) on both diets. For survival, the three independent replicates were used to obtain Kaplan Meier curves, and a log rank test was performed between both diets, with a right tailed Chi test evaluation.

### Plant Protein Inhibitor Identification

The ENSEMBL Plant genome annotations of *Z. mays* (corn) and *P. vulgaris* (green bean) were queried through BioMart (Kinsella et al., 2011) for genes associated with the GO:0030414 term “peptidase inhibition activity.” Gene IDs were recovered for each plant, and these IDs were used to retrieve their orthologs and PFAM domains from the same database. For each plant, each gene was classified according to the plants in which orthologs were found, if any, and the number of genes in each class counted. The number of PFAM domains associated with each gene in each plant was also counted and classified by the PFAM domain.

### Preparation of Protein Extracts From Tissues

For each of three biological replicates, homogenization of tissues was performed with a Polytron 2500E device (Kinematica, Luzern, Switzerland) in a 1.5 ml microcentrifuge tube on ice, using a 3:1 v/w ratio of PBS 0.1 M pH 7.4 and 10 000 rpm for 30 s. Debris was removed by two centrifugation steps at 10 000 *g*, 4°C for 10 min. Final protein concentration in the supernatant was determined by the Bradford method (Bio-Rad, Hercules, CA, United States) with BSA as a standard.

### General and Class-Specific Proteolytic Activity Assays

Proteolytic activity of midgut extracts was determined by degradation of azocasein as described previously (Lomate and Bonning, 2016) with optimization (Cantón and Bonning, 2019). In a reaction tube, 50 μg (M1) or 30 μg (M2 and M3) of tissue extract was incubated for 30 min at 37°C with or without the following inhibitors: 10 mM EDTA, 10 μM E-64, 100 μM TLCK, 100 μM TPCK, or 5 mM PMSF. The reaction was made up a to a volume of 10 μl with 0.1 M acetate buffer, pH 5.0. After incubation, 200 μl of a 1% azocasein solution in 0.1 M acetate buffer pH 5.0 was added. The tubes were then incubated at 37°C for 2 hr (M2 and M3) or overnight (M1). To stop reactions, 300 μl of chilled 5% trichloroacetic acid was added to the tubes and centrifuged for 10 min at 10 000 *g*, 4°C. In a 96-well clear bottom plate, 150 μl of 1 M NaOH was added to neutralize 150 μl of supernatant. An iD3 SpectraMax plate reader (Molecular Devices, San Jose, CA, United States) was used to measure absorbance at 450 nm.

For class-specific protease activity, 1 mM solutions of synthetic substrates were obtained by solubilizing powders in DMSO and then slowly adding 0.1 M acetate buffer pH 5.0. The final concentration of DMSO was 10% in all cases, except for pGFLpNA with a concentration of 30%. Similar to the reactions described above, 50 μg (M1) or 30 μg (M2 and M3) of midgut extract was mixed in 20 μl of acetate buffer. The reactions were incubated for 30 min at 37°C, and afterward 100 μl of substrate solution was added and again incubated at 37°C. One hundred μl of 30% acetic acid was used to stop the reaction. Absorbance was measured at 410 nm for 200 μl from each reaction in 96-well plates.

Activity units in the enzyme assays were defined as the change of 0.1 of absorbance per minute per mg of protein. Statistical comparison of equivalent treatments between diets was performed with a *t*-test with a Bonferroni correction for multiple testing after determining normality of the means of biological replicates by a Shapiro–Wilks test.

### Nucleic Acid Degradation Assays

To test nuclease activity on different substrates, we prepared 0.1 mg/ml solutions of calf thymus DNA or baker’s yeast RNA in buffer A (25 mMNaCl, 10 mM MgCl_2_, and 5 mM CaCl_2_ in a 20 mM Tris–HCl pH 8.0 nuclease-free buffer). The TranscriptAid T7 High Yield Transcription Kit (ThermoFisher, Waltham, MA, United States) was used to prepare GFP dsRNA from a 502 bp PCR template amplified from the pGlo plasmid [the primers used (Supplementary Table S1) include the T7 promoter sequence]. Dilutions of dsRNA were prepared in buffer A after purification with the PureLink RNA Mini Kit (Ambion, Foster City, CA, United States). All nucleic acid quantifications were performed by Nanodrop at 260 nm.

To monitor nuclease activity, we measured the absorbance due to the release of free nucleotides from nucleic acid substrates (Fraser, 1980). A 10 μl reaction mixture of buffer A and 10 μg of tissue extract was prepared, and then 200 μl of 0.1 mg/ml DNA or RNA in buffer A were added. Reactions were incubated for 30 min at 37°C and then stopped with 300 μl of chilled 10% trichloroacetic acid in nuclease-free water, with 20 μM sodium pyrophosphate. For dsRNA, the final reaction volume was 20 μl, with 2 μg of dsRNA and 10 μg of tissue extract in buffer A. The reactions were stopped with 30 μl of 10% trichloroacetic acid with 20 mM sodium pyrophosphate. Positive control reactions were prepared using 1 μl of 1 U/μl DNase I or 10 μg/μl RNaseA (ThermoFisher), according to substrate. Negative controls included no enzyme or extract. Once reactions were stopped, they were incubated for 1 hr on ice and then centrifuged for 10 min at 10,000 *g* and 4°C. Absorbance of the supernatant was measured at 260 nm by Nanodrop. Background absorbance of extracts was determined by following the steps above but using buffer A without substrate. We defined one unit of nuclease activity as an increase in absorbance of 0.01 per minute.

### Extraction, Purification, and Sequencing of mRNA

To purify total RNA, the PureLink RNA Mini kit (Ambion) was used. All RNAlater was removed from tissue samples, and then 600 μl of lysis buffer with β-mercaptoethanol was added. A 25G needle and syringe were used to homogenize tissues. Six hundred microliters of 70% ethanol in nuclease-free water was added and thoroughly mixed. Afterward, samples were processed according to the manufacturer’s instructions for spin columns. RNA was eluted from columns with 30 μl of nuclease-free water and quantified by Nanodrop at 260 nm. DNase I (ThermoFisher) was added to samples and incubated at 37°C for 10 min. EDTA was added to 20 mM and incubated 10 min at 65°C. Reactions were cleaned by precipitating with 100 μl of isopropanol, 5 μl of sodium acetate 3 M pH 5.2, and 2.5 μl of RNA grade glycogen and incubated overnight at −20°C. Then, samples were centrifuged at 12 000 *g*, 4°C. The pellet was washed with 70% ethanol in nuclease-free water and centrifuged at 7,600 *g* for 5 min at 4°C. The final pellet was resuspended in nuclease-free water, with concentration determined by Nanodrop. Only samples of high integrity and purity, and with at least 2 μg of total RNA were selected for RNA-Seq. Quality was reassessed at the Sequencing Facility (Supplementary Figure S1). Twenty-four RNA samples representing three biological replicates of each condition were used for the preparation of mRNA libraries that were paired-end sequenced on an Illumina HiSeq 3000 platform with 150 cycles (Genewiz, Inc., South Plainfield, NJ, United States).

### Read Processing, Assembly, and Annotation of the *de novo* Transcriptome

Reads from sequencing were processed to remove bad tiles and adapters with FilterByTile (BBMap Suite (Bushnell, 2015) parameters: *d* = 0.75, *qd* = 1, *ed* = 1, *va* = 0.5, *qa* = 0.5, *ea* = 0.5) and TrimGalore v 0.4.4 (Krueger, 2019) (parameters: - length 36, - q 5, - stringency 1, -e 0.1), respectively. Two complete sets of duplicates of the four tissues analyzed from corn and green bean diet samples were pooled to perform *de novo* transcriptome assembly with Trinity 2.8.3 [(Grabherr et al., 2011), - normalize_max_read_cov 200 and - min_kmer_cov 2]. Transcript abundance for all tissues in triplicate was obtained with Salmon v 0.12 (Patro et al., 2017) with the –gcBiasflag. N50, ExN50, and transcripts per kilobase million (TPM) values for the transcripts in the assembly were obtained using scripts in the Trinity package and the Salmon abundance files. Completeness of transcript recovery was evaluated with BUSCO v3.01 (Waterhouse et al., 2017) with reference to the arthropod ortholog dataset. To annotate the transcriptome, we followed the online vignette for Trinotate v3.0.1 (Haas et al., 2013).

### Differential Expression Analysis for Assembled Transcripts and Functional Enrichment

The BioconductoR package TxImport 1.8.0 (Soneson et al., 2015) was used to import transcript abundance data from the quant.sf files of the three biological replicates of each diet using the lengthScaledTPM and dropInfReps = TRUE parameters. The txOut = TRUE argument was used to retain abundance data at the transcript level. DESeq2 v 1.20.0 (Love et al., 2014) was used to perform the statistical analysis of differential expression. Statistical testing incorporated the fold change shrinkage with the apeglm algorithm (Zhu et al., 2018) and a fold change threshold above 1 or below −1. Cutoff for significance was set at an *s*-value of 0.005. DESeq2 and ggplot2 were used to prepare PCA plots. The Trinotate annotation information was filtered using the IDs of differentially transcribed sequences for each comparison between corn and green bean tissues. Transcripts with a BLASTp, BLASTx, or PFAM match were counted. A custom R script was written to identify IDs of statistically significant transcripts that were common to lists from the comparisons of salivary glands, M1, M2, and M3. TPM from all samples was recovered for transcripts that had a significant fold change in two or more comparisons.

Enrichment of GO terms was determined for transcripts with significant differences using the TopGO package (Alexa and Rahnenfuhrer, 2018). For this, the Trinotate results for GO terms inferred from BLAST and PFAM hits were used to create a custom GO annotation reference with rows containing the transcript ID and all associated GO terms. TopGO was used to build a GO graph object with annotated lists of significantly different transcripts and the custom annotation file. A node size of five or higher was used as cutoff for terms included in statistical testing. Terms in the “Molecular Function” topology were evaluated with the “weight” algorithm (Alexa et al., 2006). A *p*-value of 0.05 established as cutoff for the Fisher exact test.

## Results

### Impacts of Diet Type in Stink Bug Growth

**FIGURE 1:**
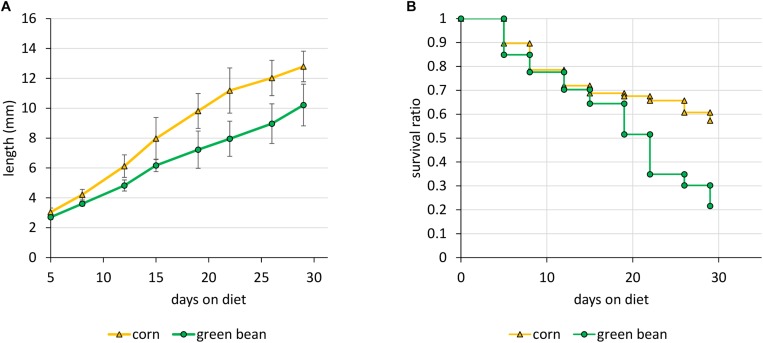
Growth and survival of *N. viridula* on an exclusive diet of corn or green bean. Parameters were measured starting with forty 2nd instar nymphs. **(A)** Growth of surviving individuals. **(B)** Survival ratio. Error bars represent SEM of three biological replicates. Differences in growth and survival were both significant, with *p*-values of < 0.01, by a *t*-test of linear regression for length and a log rank test for survival.

In order to explore the effects of specific diet types on the physiology of *N. viridula*, we first reared nymphs exclusively on either a graminoid (corn, *Z. mays*) or a legume (green bean, *P. vulgaris*). We monitored the growth and survival of the nymphs throughout their development. Under our rearing conditions, nymphs performed better on a diet of corncob with kernels than on green bean pods. Although both types of diet allowed some nymphs to molt into adults, green bean fed nymph growth lagged behind those fed on corn, and survival was higher throughout on corn (Figure 1). Differences in growth were significant (*p*-value < 0.0005), as were those in survival (*p*-value of < 0.009).

Plants produce protease inhibitors as a defense mechanism against herbivory (Zhu-Salzman and Zeng, 2015). We queried the publicly available annotation for corn and green bean to determine the differences in their repertoire of protease inhibitors. We identified 83 and 41 genes corresponding to the “peptidase inhibition activity” molecular function GO annotation for corn and green bean, respectively. However, only eight (9.6%, corn) or seven (17%, green bean) of the protease inhibitors genes were orthologs between the two plants (Supplementary Figure S2A). Additionally, we analyzed the PFAM domains for these genes where available. For both plants, the identified domains were mostly comprised by serine protease inhibitors, with around 20% of the genes corresponding to cysteine protease inhibitors (Supplementary Figure S2B). Among the serine protease inhibitor domains, in green bean the majority were identified as belonging to Kunitz STI type protein inhibitors, while in corn the largest proportion of PFAM domains belonged to the potato inhibitor family I.

### Comparison of Digestive Enzyme Activity Between Corn and Green Bean Diets

**FIGURE 2:**
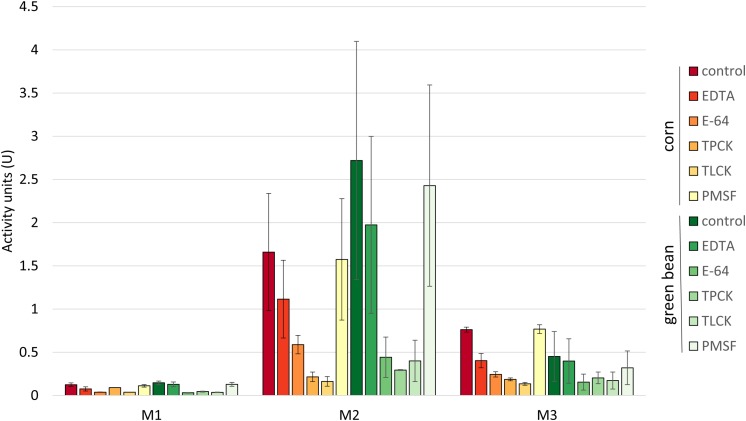
Proteolytic profiles of midgut tissues are similar for insects fed on corn or green bean diet. Activity units for degradation of azocasein were compared between each inhibitor treatment of the corresponding tissue extract from the two diets. A *t*-test with a Bonferroni correction for multiple testing with α = 0.5 was used. No significant differences were found. Error bars represent SEM of three biological replicates.

We assessed whether the differences in stink bug growth on the two diets would be reflected by changes in digestive enzyme activity. The digestive enzyme activity of protein extracts from M1, M2, and M3 tissues of *N. viridula* adults grown on green bean diet was compared to profiles previously determined for the same tissues from corn fed *N. viridula* (Cantón and Bonning, 2019). First, we tested the protein extracts from the M1, M2, and M3 midgut regions for proteolytic activity on an azocasein substrate. The overall activity profile and sensitivity to inhibitors was similar for both diets, with no significant differences between corresponding tissue assays (Figure 2). On both diets most proteolytic activity was detected in M2 extracts, followed by M3, with the lowest activity in M1. The inhibitors EDTA and PMSF had no effect on proteolytic activity in M2 for either diet.

**FIGURE 3:**
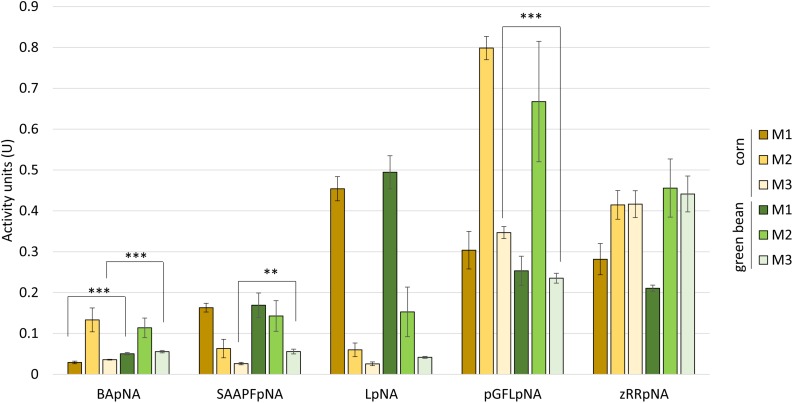
Protease class-specific activity does not differ greatly between corn and green bean diet. Synthetic substrates class: trypsin (BApNA), chymotrypsin (SAAPFpNA), aminopeptidase (LpNA), cysteine proteases (pGFLpNA), and cathepsin B (zRRpNA). A *t*-test with a Bonferroni correction for multiple testing with α = 0.5 was used to compare between corresponding substrate/tissue assays within each substrate. ^∗∗^ *p*-value < 0.05, ^∗∗∗^ *p*-value < 0.01. Error bars represent SEM of three biological replicates.

We then used class-specific substrates to more precisely determine the type of proteases active in each of the extracts (Figure 3). As for the azocasein activity assays, no significant differences between the two diets were seen for most comparisons of the corresponding M1, M2, or M3 extracts. The most significant differences were observed for the degradation of the trypsin substrate BApNA, where the green bean diet extracts for M1 and M3 showed higher activity than the corn diet extracts (Figure 3). The lower degradation of the cysteine protease substrate pGFLpNA by extracts from M3 of green bean-fed compared to corn-fed stink bugs was also highly significant. The overall profile of substrate degradation was similar between the two diets for the remaining substrates.

**FIGURE 4:**
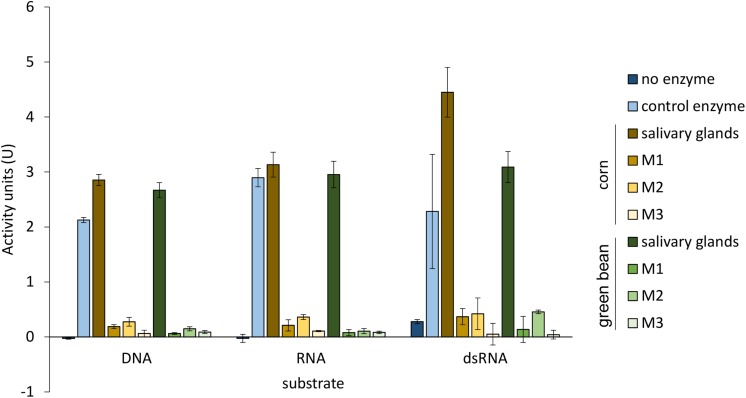
Nuclease activity is comparable between corresponding tissues from corn and green bean diet. DNase I and RNase A were used as positive control enzymes for DNA substrate and RNA and dsRNA substrates, respectively. Differences in nuclease activity between corresponding tissue extracts from each diet with each substrate were determined by a *t*-test with Bonferroni correction of α = 0.5. Error bars represent SEM of three biological replicates.

The activity profiles for nucleases in the midgut and the salivary glands did not differ between the two diets for any of the tissue extracts analyzed (Figure 4). As reported previously (Lomate and Bonning, 2016), salivary glands showed high nuclease and ribonuclease activity compared to the whole midgut. Data for the individually analyzed midgut regions were consistent with this result.

### Impact of Diet on Transcription

**FIGURE 5:**
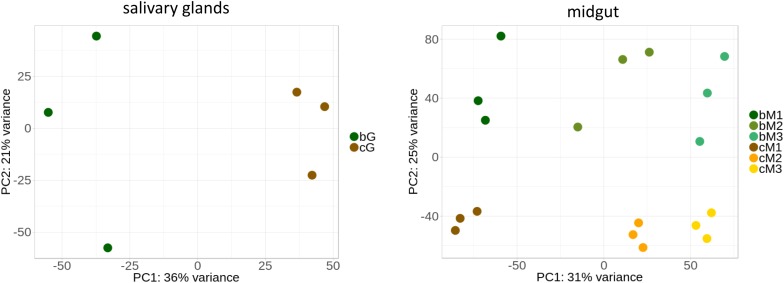
Principal component analysis can distinguish between corn and green bean diet samples. Salivary gland and midgut regions were analyzed separately to avoid masking of diet-derived differences (see text). B = green bean, C = corn, G = salivary glands. Three biological replicates were obtained for each condition.

Although few significant differences were detected in enzyme activities between the two diets, we hypothesized that regulation at the transcriptional level could drive diet-related compensatory changes. We therefore compared the transcriptomes between stink bugs fed on the two diets. We performed RNA-seq on the salivary glands, M1, M2, and M3 of adults grown on green bean diet and compared these results to our previous transcriptomic dataset of adult *N. viridula* fed on corn (Cantón and Bonning, 2019). We tested for differential expression using separate sets of samples for the salivary glands and the midgut regions. Figure 5 shows the principal component analysis of these two datasets. For all tissues, the corn and green bean samples cluster separately, indicating that differences in transcript abundance between diet types are distinct. The degree of separation of clusters for each midgut region for the two diets is comparable. The dispersion is notably higher for data from insects fed green bean compared to those fed corn, with the widest dispersion seen for salivary gland samples.

Table 1 summarizes the number of transcripts from each of the comparisons between matching tissues in corn- and green bean-fed insects, using the corresponding green bean-fed sample as reference. M2 had the most transcripts with a significant fold change with 244, while M3 had the least. Overall, pairwise comparisons showed that more transcripts were upregulated in corn than downregulated. We attempted GO enrichment analysis for the upregulated and downregulated transcripts, but a large number corresponded to sequences that could not be annotated through Trinotate. This led to unclear results with only a small number of transcripts per category (Supplementary File S1). In light of this, we identified transcripts that had a significant fold change in more than one pairwise comparison as potential diet specific transcripts regardless of annotation. By this method, we created sets of transcripts whose induction is more related to corn or green bean feeding (downregulation in corn being relative to upregulation in green bean). These results are summarized in Table 2. One transcript was significantly upregulated in all corn tissues, TRINITY_DN1479_c0_g1, which encodes a serine protease. The unannotated transcript TRINITY_DN1811_c2_g1_i8 was downregulated in corn for three pairwise comparisons. In general, few of these transcripts with significant fold changes could be annotated through homology or PFAM domain identification.

**TABLE 1:** Numbers of significantly upregulated or downregulated transcripts between tissues from insects fed on corn or green bean diets.

**Tissue**	**Total**	**Upregulated**	**Downregulated**	**Analyzed**
Salivary glands	123	110	13	186378
M1	124	100	24	281989
M2	244	156	88	281989
M3	35	32	3	281989

*In each comparison, the corresponding tissue in the green bean diet samples was set as reference. The number of analyzed transcripts is those that passed the filtering step of DEseq2.*

**TABLE 2:** Number of unique transcripts with significant fold change between corn and green bean diet in more than one tissue.

**Number of comparisons**	**Number of transcripts**	**Annotated**	**Unannotated**
**Upregulated**			
1	309	153	156
2	32	14	18
3	7	1	6
4	1	1	0
**Downregulated**			
1	101	67	34
2	12	4	8
3	1	0	1
4	0	–	–

*In each comparison, the corresponding tissue in the green bean diet samples was set as reference.*

**FIGURE 6:**
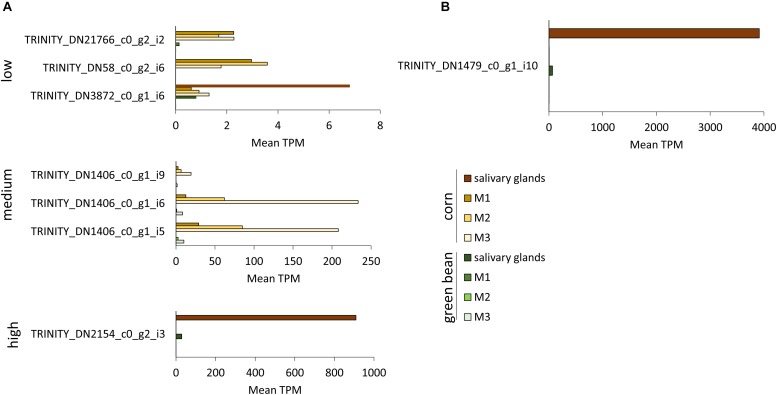
TPM of transcripts significantly upregulated in three or more tissues from corn-fed insects. TPM values were calculated with the Trinity suite. **(A)** Transcripts with significant differences in three pairwise comparisons. **(B)** Transcripts with significant differences in all pairwise comparisons.

**FIGURE 7:**
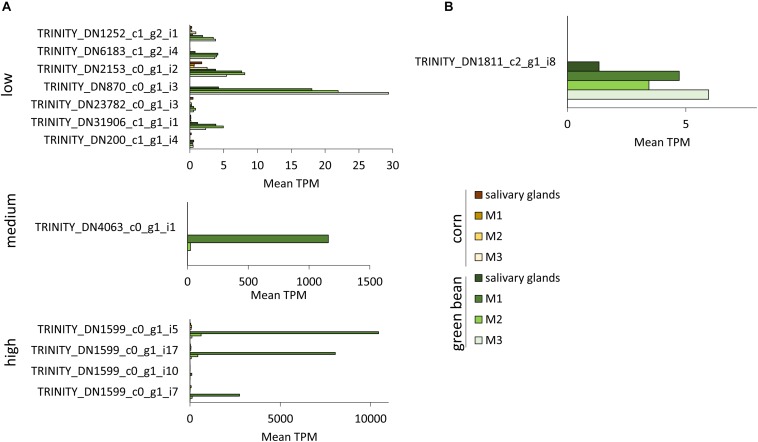
TPM of transcripts significantly downregulated in two or three tissues from corn-fed insects. TPM values were calculated with Trinity suite. **(A)** Transcripts with significant differences in two pairwise comparisons. **(B)** Transcripts with significant differences in three pairwise comparisons.

Transcripts with significant fold-change in more than one comparison were separated into low, medium, and high TPM transcripts (Figures 6, 7 and Supplementary Figure S3). TRINITY_DN3872_c0_g1_i6, TRINITY_DN58_c0_g2_i6, and TRINITY_DN21766_c0_g2_i2 all had significant upregulation in three comparisons, but their TPM was below 10 in all tissues (Figure 6A). The transcripts with medium TPM that were significantly upregulated in corn in three comparisons correspond to isoforms of TRINITY_DN1406_c0_g1, with higher TPM in M3 tissue. Although these transcripts are unannotated they do contain a predicted secretion signal peptide. The unannotated transcript TRINITY_DN2154_c0_g2_i3 is significantly upregulated in corn in three comparisons, with very high TPM in salivary glands and very low TPM in the three midgut regions (although higher than the corresponding green bean-fed samples). A similar profile can be seen for the upregulated serine protease significant in four comparisons (Figure 6B). Transcript TRINITY_DN870_c0_g1 has homology to Hrp65, a protein involved in RNA maturation. This transcript is significantly downregulated in corn compared to green bean in two comparisons, although the TPM is higher in all green bean tissues (Figure 6A). Of note is the very high TPM in green bean tissues of isoforms of TRINITY_DN1599_c0_g1, particularly for M1. This transcript is unannotated but has a secretion signal peptide (Figure 7A). Finally, TRINITY_DN1811_c2_g1, which was significantly downregulated in corn for three comparisons, has a low TPM in all green bean tissues, but was not transcribed in any of the corn samples (Figure 7B).

## Discussion

We sought to determine if and how the diet could change the digestive physiology of *N. viridula* toward improved understanding of *N. viridula* polyphagy. No major differences were detected by enzyme assay of digestive tissues from insects maintained on corn or green bean diets in protease inhibitor sensitivity, protease class profiles, or nuclease activity between any of the tissues analyzed. Although no single gene or category of genes was wholly responsible for the clustering of samples by diet type observed in our PCA plots, these results indicate that there is a measurable, diet-specific transcriptomic change. Some specific transcripts show significant differences between the diets, but many could not be assigned an annotation. Further assessment is needed to assign these diet-related, differentially transcribed genes to specific functions.

Differential regulation of insect gene expression has been noted in response to host plant composition and defenses against herbivory. Changes in the amount and type of protease expressed have been observed for several insects (Jongsma et al., 1995; Moon et al., 2004; Brioschi et al., 2007; de Oliveira et al., 2013; Fescemyer et al., 2013; Rivera-Vega et al., 2017), typically with a shift to enzymes less sensitive to the inhibitory compounds present in the diet. Other diet-induced changes involve the active degradation of anti-nutritional factors that might otherwise limit nutrition (Girard et al., 1998). The response to host plant is not limited to digestive enzymes, but involves other metabolic processes including detoxification, stress response, and immunity pathways (Huang et al., 2017). However, diet-dependent changes in transcription in hemipteran species may not be as pronounced or even involve digestive enzymes. Feeding on diets with different phytotoxins only induced changes in a few detoxification enzymes in *Bemisia tabaci* (Halon et al., 2015). For the scale insect *Paratachardina pseudolobata*, feeding on different host plants induced transcriptional changes, yet only a fraction of the genes was related to detoxification, being otherwise enriched for primary metabolism functions (Christodoulides et al., 2017). In the pea aphid *Acyrthosiphon pisum*, changes in transcription were more pronounced for race effect than host plant effect, with only a few hundred genes having expression changes attributable to diet type (Eyres et al., 2016). More recently, in the closely related stink bug *Halyomorpha halys*, no significant changes were detected on different host plants for cytochrome P450 enzymes involved in detoxification (Mittapelly et al., 2019). Our results of a somewhat moderate response in transcriptional changes after feeding on different diets are therefore not entirely unexpected and are similar to those observed for other hemipterans, in contrast to clear responses reported for lepidopteran and coleopteran species. It has been proposed that the feeding mechanism (i.e., sucking vs. chewing) could be an important factor in determining the elicited plant responses to herbivory (Ali and Agrawal, 2012), and consequently in the compensatory mechanisms that the insects have evolved (Will et al., 2013).

One consideration in the evolution of a diet dependent response is that adaptation to host plant composition requires resources that would otherwise be allocated to growth and development. In the Colorado potato beetle, the presence of Cathepsin D inhibitor in plant diet was accompanied by initial effects on growth (Brunelle et al., 2004). Another coleopteran, the cowpea bruchid, also showed reduced early instar growth when fed on diet with the soyacystatin inhibitor, but resumed normal development at the 4th instar (Moon et al., 2004). Despite a preference for legumes, in our experiments the development of *N. viridula* fed on green beans was delayed. The total nymphal survival was slightly below that reported for this species when grown exclusively on green bean pods (Panizzi and Slansky, 1991). We cannot rule out that as our test population was reared under laboratory conditions for several generations it had lost tolerance for this food source. Additionally, regional variation in survivorship has been detected (Panizzi, 1997), and the origin of the initial colony (Louisiana) and supplementation with individuals from Florida may have contributed to this effect. While insects in this study were fed on corncob and green beans which were detached from the plants, additional responses may occur when insects are fed on live plants. In this scenario, however, responses to differing diet may be confounded by insect responses to induced plant defenses.

For *N. viridula*, most proteolytic digestion will take place in M2 and M3. In M2, digestion is mediated by cysteine proteases, such as Cathepsin B and L (Cantón and Bonning, 2019). Analysis of inhibitor domains indicated that while both green bean and corn had a similar number of cystatins, green beans have more Kunitz-type inhibitors than corn. These inhibitors generally target serine proteases, although at least one cysteine protease inhibitor in plants has been reported with a Kunitz-type domain (Rustgi et al., 2018). If Kunitz-type inhibitors from green bean inhibit cysteine proteases, this could contribute to the inferior nutritional state of insects maintained on green bean relative to corn. Although no changes in inhibitor sensitivity were detected in adults, nor specific cysteine proteases upregulated in green beans, other compensatory functions that deal with what appears to a be more difficult source of nutrition may be identified from the unannotated transcripts with higher transcription in green beans (such as TRINITY_DN1811_c2_g1_i8). Although legume seeds can have high levels of trypsin inhibitors (El-Morsi, 2001), the low activity of this enzyme class in the midgut tissues (as observed in this work) is characteristic of the hemipteran lineage (Terra and Ferreira, 1994). These inhibitors are less likely to have an effect on *N. viridula* digestion.

The biological bases for how generalist insects safely ingest a wide variety of plant compounds is highly relevant in the context of plant-insect interactions for pests that feed on multiple crops of economic interest. The work presented here improves our understanding of the physiological responses of the digestive tract of *N. viridula* when feeding on legume and graminoid diets, and provides leads for future investigation of the role of the differentially regulated, unannotated transcripts in adaptation to host plant.

## Data Availability Statement

Raw sequence reads from this project can be accessed at NCBI Short Read Archive SRP193118. Transcript assembly and abundance tables are available at NCBI Gene Expression Omnibus ID GSE130097.

## Author Contributions

PC performed the experiments and bioinformatics analysis. PC and BB devised the experiments and wrote the manuscript.

## Conflict of Interest

The authors declare that the research was conducted in the absence of any commercial or financial relationships that could be construed as a potential conflict of interest.
