# Supplementary material for: Transcription and Activity of Digestive Enzymes of Nezara viridula Maintained on Different Plant Diets
Source: Front Physiol. 2020 Jan 8;10:1553. doi: 10.3389/fphys.2019.01553 (PMC6960134; doi:10.3389/fphys.2019.01553)
Supplement: Supplementary file 1 [file Data_Sheet_1.PDF]

Upregulated and downregulated transcripts of *N. viridula* fed on corn or green bean diet, by tissue: salivary gland and gut regions, M1, M2 and M3.

Corn gland vs green bean gland: upregulated

GO.ID	Term	Annotated	Significant	Expected	classicFisher
GO:0004252	serine-type endopeptidase activity	366	8	0.52	4.7e-08
GO:0070330	aromatase activity	84	3	0.12	0.00023
GO:0005089	Rho guanyl-nucleotide exchange factor activity	151	3	0.21	0.00129
GO:0019894	kinesin binding	59	2	0.08	0.00318
GO:0004714	transmembrane receptor protein tyrosine kinase activity	65	2	0.09	0.00384
GO:0008083	growth factor activity	73	2	0.1	0.00482
GO:0001855	complement component C4b binding	5	1	0.01	0.00704
GO:0016015	morphogen activity	5	1	0.01	0.00704
GO:0005524	ATP binding	5006	14	7.06	0.00842
GO:0000026	alpha-1,2-mannosyltransferase activity	7	1	0.01	0.00984
GO:0035325	Toll-like receptor binding	7	1	0.01	0.00984
GO:0004376	glycolipid mannosyltransferase activity	7	1	0.01	0.00984
GO:0004341	gluconolactonase activity	8	1	0.01	0.01123
GO:0004719	protein-L-isoaspartate (D-aspartate) O-methyltransferase activity	9	1	0.01	0.01263
GO:1990599	3' overhang single-stranded DNA endodeoxyribonuclease activity	10	1	0.01	0.01402
GO:0005121	Toll binding	11	1	0.02	0.01542

GO:0051059	NF-kappaB binding	11	1	0.02	0.01542
GO:0071933	Arp2/3 complex binding	12	1	0.02	0.01681
GO:0001026	TFIIIB-type transcription factor activity	12	1	0.02	0.01681
GO:0043560	insulin receptor substrate binding	13	1	0.02	0.01819
GO:0008821	crossover junction endodeoxyribonuclease activity	13	1	0.02	0.01819
GO:0042834	peptidoglycan binding	14	1	0.02	0.01958
GO:0034452	dynactin binding	15	1	0.02	0.02096
GO:0004816	asparagine-tRNA ligase activity	16	1	0.02	0.02235
GO:0005001	transmembrane receptor protein tyrosine phosphatase activity	16	1	0.02	0.02235
GO:0043539	protein serine/threonine kinase activator activity	16	1	0.02	0.02235
GO:0034236	protein kinase A catalytic subunit binding	17	1	0.02	0.02373
GO:0001094	TFIID-class transcription factor binding	17	1	0.02	0.02373
GO:0008294	calcium- and calmodulin-responsive adenylate cyclase activity	18	1	0.03	0.02510
GO:0004555	alpha,alpha-trehalase activity	18	1	0.03	0.02510
GO:0004176	ATP-dependent peptidase activity	22	1	0.03	0.03060
GO:0001156	TFIIIC-class transcription factor binding	22	1	0.03	0.03060
GO:0008329	signaling pattern recognition receptor activity	23	1	0.03	0.03197
GO:0005096	GTPase activator activity	493	3	0.7	0.03253
GO:0000150	recombinase activity	24	1	0.03	0.03333
GO:0032183	SUMO binding	24	1	0.03	0.03333

GO:0008094	DNA-dependent ATPase activity	211	2	0.3	0.03591
GO:0005506	iron ion binding	515	3	0.73	0.03630
GO:0050321	tau-protein kinase activity	27	1	0.04	0.03742
GO:0020037	heme binding	525	3	0.74	0.03809
GO:0005085	guanyl-nucleotide exchange factor activity	454	5	0.64	0.04050
GO:0043559	insulin binding	31	1	0.04	0.04285
GO:0008353	RNA polymerase II carboxy-terminal domain kinase activity	32	1	0.05	0.04420
GO:0043548	phosphatidylinositol 3-kinase binding	35	1	0.05	0.04824

Corn gland vs green bean gland: downregulated

**GO.ID**	**Term**	**Annotated**	**Significant**	**Expected**	**classicFisher**
GO:0016985	mannan endo-1,4-beta-mannosidase activity	64	2	0.01	3.8e-05
GO:0034189	very-low-density lipoprotein particle binding	5	1	0	0.00076
GO:0038025	reelin receptor activity	5	1	0	0.00076
GO:0030229	very-low-density lipoprotein particle receptor activity	5	1	0	0.00076
GO:0034437	glycoprotein transporter activity	22	1	0	0.00332
GO:0008474	palmitoyl-(protein) hydrolase activity	38	1	0.01	0.00573
GO:0034185	apolipoprotein binding	42	1	0.01	0.00633
GO:0048306	calcium-dependent protein binding	55	1	0.01	0.00829

Corn M1 vs green bean M1: upregulated

GO.ID	Term	Annotated	Significant	Expected	classicFisher
GO:0004252	serine-type endopeptidase activity	366	7	0.4	1.3e-07
GO:0008234	cysteine-type peptidase activity	576	6	0.62	3.5e-05
GO:0008465	glycerate dehydrogenase activity	13	2	0.01	8.9e-05
GO:0016618	hydroxypyruvate reductase activity	23	2	0.02	0.00029
GO:0030267	glyoxylate reductase (NADP) activity	23	2	0.02	0.00029
GO:0070402	NADPH binding	75	2	0.08	0.00303
GO:0051287	NAD binding	269	3	0.29	0.00311
GO:0052689	carboxylic ester hydrolase activity	636	4	0.69	0.00492
GO:0015180	L-alanine transmembrane transporter activity	6	1	0.01	0.00648
GO:0015193	L-proline transmembrane transporter activity	6	1	0.01	0.00648
GO:0005199	structural constituent of cell wall	8	1	0.01	0.00864
GO:0008477	purine nucleosidase activity	9	1	0.01	0.00971
GO:0045437	uridine nucleosidase activity	9	1	0.01	0.00971
GO:0004008	copper-exporting ATPase activity	9	1	0.01	0.00971
GO:0043023	ribosomal large subunit binding	10	1	0.01	0.01078
GO:1903136	cuprous ion binding	10	1	0.01	0.01078
GO:0030984	kininogen binding	11	1	0.01	0.01186
GO:0015574	trehalose transmembrane transporter activity	157	2	0.17	0.01263

GO:0005415	nucleoside:sodium symporter activity	12	1	0.01	0.01293
GO:0047134	protein-disulfide reductase activity	16	1	0.02	0.01720
GO:0016532	superoxide dismutase copper chaperone activity	17	1	0.02	0.01827
GO:0031406	carboxylic acid binding	206	2	0.22	0.02106
GO:0032767	copper-dependent protein binding	22	1	0.02	0.02358
GO:0015173	aromatic amino acid transmembrane transporter activity	22	1	0.02	0.02358
GO:0004021	L-alanine:2-oxoglutarate aminotransferase activity	37	1	0.04	0.03934
GO:0003857	3-hydroxyacyl-CoA dehydrogenase activity	41	1	0.04	0.04350
GO:0000182	rDNA binding	45	1	0.05	0.04764

Corn M1 vs green bean M1: downregulated

GO.ID	Term	Annotated	Significant	Expected	classicFisher
GO:0004062	aryl sulfotransferase activity	24	1	0	0.0042
GO:0046912	transferase activity, transferring acyl groups, acyl groups converted into alkyl on transfer	27	1	0	0.0048
GO:0050840	extracellular matrix binding	34	1	0.01	0.0060
GO:0070403	NAD+ binding	86	1	0.02	0.0151
GO:0004725	protein tyrosine phosphatase activity	170	1	0.03	0.0296
GO:0004004	ATP-dependent RNA helicase activity	225	1	0.04	0.0390

Corn M2 vs green bean M2: upregulated

GO.ID	Term	Annotated	Significant	Expected	classicFisher
GO:0004252	serine-type endopeptidase activity	366	7	0.53	1.1e-06
GO:0043178	alcohol binding	84	3	0.12	0.00026
GO:0032453	histone demethylase activity (H3-K4 specific)	18	2	0.03	0.00032
GO:0051864	histone demethylase activity (H3-K36 specific)	22	2	0.03	0.00048
GO:0071949	FAD binding	73	2	0.11	0.00516
GO:0005549	odorant binding	78	2	0.11	0.00587
GO:0016015	morphogen activity	5	1	0.01	0.00729
GO:0008431	vitamin E binding	6	1	0.01	0.00874
GO:0045437	uridine nucleosidase activity	9	1	0.01	0.01308
GO:0070122	isopeptidase activity	9	1	0.01	0.01308
GO:0008477	purine nucleosidase activity	9	1	0.01	0.01308
GO:0004180	carboxypeptidase activity	127	2	0.19	0.01494
GO:0015125	bile acid transmembrane transporter activity	11	1	0.02	0.01596
GO:0005121	Toll binding	11	1	0.02	0.01596
GO:0120013	intermembrane lipid transfer activity	13	1	0.02	0.01884
GO:0004731	purine-nucleoside phosphorylase activity	14	1	0.02	0.02027
GO:0103068	leukotriene C4 gamma-glutamyl transferase activity	14	1	0.02	0.02027
GO:0008241	peptidyl-dipeptidase activity	14	1	0.02	0.02027

GO:0102953	hypoglycin A gamma-glutamyl transpeptidase activity	14	1	0.02	0.02027
GO:0003997	acyl-CoA oxidase activity	14	1	0.02	0.02027
GO:0036374	glutathione hydrolase activity	14	1	0.02	0.02027
GO:0034235	GPI anchor binding	17	1	0.02	0.02456
GO:0004165	dodecenoyl-CoA delta-isomerase activity	18	1	0.03	0.02599
GO:0004555	alpha,alpha-trehalase activity	18	1	0.03	0.02599
GO:0004647	phosphoserine phosphatase activity	19	1	0.03	0.02741
GO:0016842	amidine-lyase activity	21	1	0.03	0.03026
GO:0102549	1-18:1-2-16:0-monogalactosyldiacylglycerol lipase activity	23	1	0.03	0.03309
GO:0047714	galactolipase activity	23	1	0.03	0.03309
GO:0001618	virus receptor activity	23	1	0.03	0.03309
GO:0031957	very long-chain fatty acid-CoA ligase activity	24	1	0.04	0.03450
GO:0098519	nucleotide phosphatase activity, acting on free nucleotides	24	1	0.04	0.03450
GO:0043325	phosphatidylinositol-3,4-bisphosphate binding	25	1	0.04	0.03592
GO:0033883	pyridoxal phosphatase activity	27	1	0.04	0.03873
GO:0008239	dipeptidyl-peptidase activity	27	1	0.04	0.03873
GO:0005506	iron ion binding	515	3	0.75	0.03966
GO:0003785	actin monomer binding	28	1	0.04	0.04014

Corn M2 vs green bean M2: downregulated

GO.ID	Term	Annotated	Significant	Expected	classicFisher
GO:0047988	hydroxyacid-oxoacid transhydrogenase activity	8	2	0.01	4.7e-05
GO:0043531	ADP binding	52	2	0.07	0.0021
GO:0070330	aromatase activity	84	2	0.11	0.0055
GO:0004342	glucosamine-6-phosphate deaminase activity	7	1	0.01	0.0091
GO:0042799	histone methyltransferase activity (H4-K20 specific)	8	1	0.01	0.0104
GO:0016040	glutamate synthase (NADH) activity	8	1	0.01	0.0104
GO:0098808	mRNA cap binding	8	1	0.01	0.0104
GO:0015349	thyroid hormone transmembrane transporter activity	11	1	0.01	0.0143
GO:0050354	triokinase activity	14	1	0.02	0.0182
GO:0102571	[protein]-3-O-(N-acetyl-D-glucosaminyl)-L-serine/L-threonine O-N-acetyl-alpha-D-glucosaminase activity	14	1	0.02	0.0182
GO:0034012	FAD-AMP lyase (cyclizing) activity	14	1	0.02	0.0182
GO:0102166	[protein]-3-O-(N-acetyl-D-glucosaminyl)-L-threonine O-N-acetyl-alpha-D-glucosaminase activity	14	1	0.02	0.0182
GO:0102167	[protein]-3-O-(N-acetyl-D-glucosaminyl)-L-serine O-N-acetyl-alpha-D-glucosaminase activity	14	1	0.02	0.0182
GO:0008273	calcium, potassium:sodium antiporter activity	15	1	0.02	0.0195
GO:0016231	beta-N-acetylglucosaminidase activity	16	1	0.02	0.0208
GO:0051538	3 iron, 4 sulfur cluster binding	16	1	0.02	0.0208
GO:0004371	glycerone kinase activity	17	1	0.02	0.0220

GO:0061629	RNA polymerase II sequence-specific DNA binding transcription factor binding	17	1	0.02	0.0220
GO:0004022	alcohol dehydrogenase (NAD) activity	18	1	0.02	0.0233
GO:0017070	U6 snRNA binding	19	1	0.02	0.0246
GO:0003917	DNA topoisomerase type I activity	21	1	0.03	0.0272
GO:0043015	gamma-tubulin binding	21	1	0.03	0.0272
GO:0004559	alpha-mannosidase activity	21	1	0.03	0.0272
GO:0005506	iron ion binding	515	3	0.67	0.0300
GO:0031402	sodium ion binding	25	1	0.03	0.0323
GO:0003724	RNA helicase activity	235	2	0.31	0.0381
GO:0004556	alpha-amylase activity	30	1	0.04	0.0386
GO:0103025	alpha-amylase activity (releasing maltohexaose)	30	1	0.04	0.0386
GO:0046982	protein heterodimerization activity	582	3	0.76	0.0408
GO:0050840	extracellular matrix binding	34	1	0.04	0.0436
GO:0035255	ionotropic glutamate receptor binding	35	1	0.05	0.0449
GO:0070530	K63-linked polyubiquitin modification-dependent protein binding	36	1	0.05	0.0461
GO:0044183	protein binding involved in protein folding	37	1	0.05	0.0474
GO:0030955	potassium ion binding	38	1	0.05	0.0486
GO:0050681	androgen receptor binding	38	1	0.05	0.0486
GO:0015347	sodium-independent organic anion transmembrane transporter activity	39	1	0.05	0.0499

Corn M3 vs green bean M3: upregulated

GO.ID	Term	Annotated	Significant	Expected	classicFisher
GO:0019531	oxalate transmembrane transporter activity	30	2	0.01	3.6e-05
GO:0008271	secondary active sulfate transmembrane transporter activity	32	2	0.01	4.1e-05
GO:0015106	bicarbonate transmembrane transporter activity	35	2	0.01	5.0e-05
GO:0015301	anion:anion antiporter activity	61	2	0.02	0.00015
GO:0003993	acid phosphatase activity	62	2	0.02	0.00016
GO:0005254	chloride channel activity	139	2	0.04	0.00079
GO:0004008	copper-exporting ATPase activity	9	1	0	0.00272
GO:1903136	cuprous ion binding	10	1	0	0.00302
GO:0016532	superoxide dismutase copper chaperone activity	17	1	0.01	0.00513
GO:0004252	serine-type endopeptidase activity	366	2	0.11	0.00527
GO:0016842	amidine-lyase activity	21	1	0.01	0.00633
GO:0032767	copper-dependent protein binding	22	1	0.01	0.00663
GO:0005521	lamin binding	43	1	0.01	0.01293
GO:0048365	Rac GTPase binding	98	1	0.03	0.02924
GO:0003705	transcription factor activity, RNA polymerase II distal enhancer sequence-specific binding	125	1	0.04	0.03716
GO:0051087	chaperone binding	153	1	0.05	0.04530

Corn M3 vs green bean M3: downregulated

GO.ID	Term	Annotated	Significant	Expected	classicFisher
GO:0008201	heparin binding	98	1	0	0.0049
GO:0004620	phospholipase activity	155	1	0.01	0.0078
GO:0052689	carboxylic ester hydrolase activity	636	1	0.03	0.0318
