# Supplementary figures and images for: Transcription and Activity of Digestive Enzymes of Nezara viridula Maintained on Different Plant Diets
Source: Front Physiol. 2020 Jan 8;10:1553. doi: 10.3389/fphys.2019.01553 (PMC6960134; doi:10.3389/fphys.2019.01553)

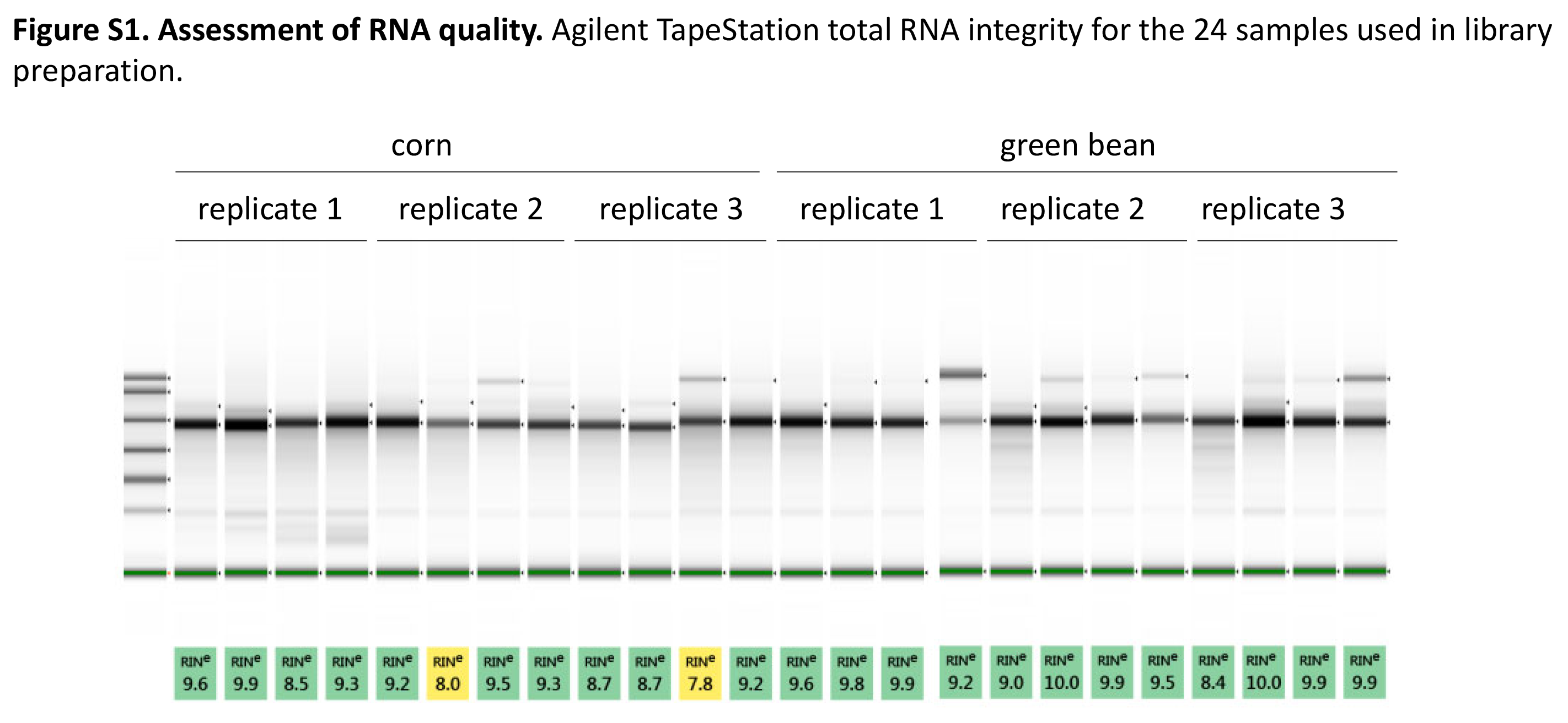

Supplement: Supplementary file 2 [file Image_1.TIF]

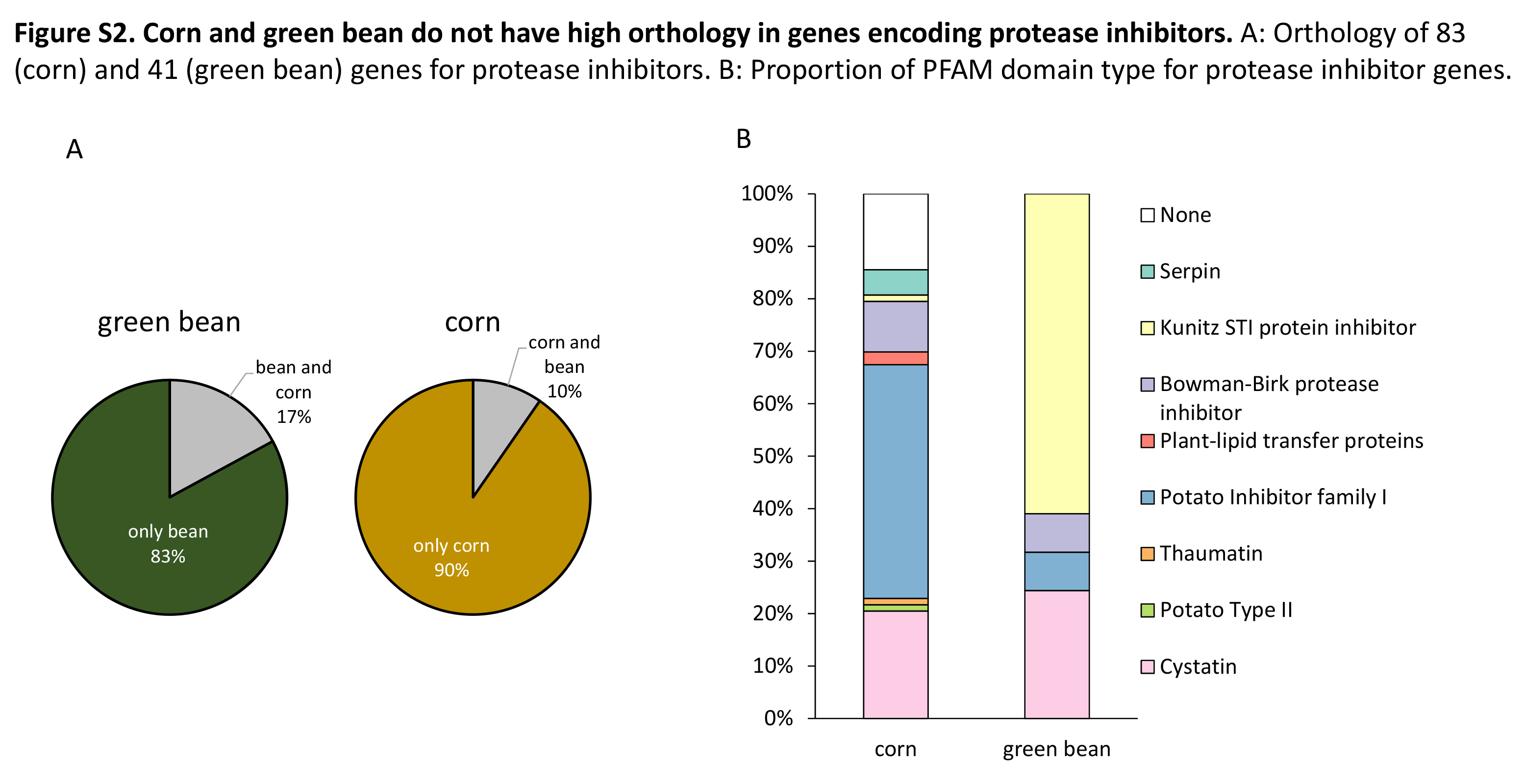

Supplement: Supplementary file 3 [file Image_2.TIF]

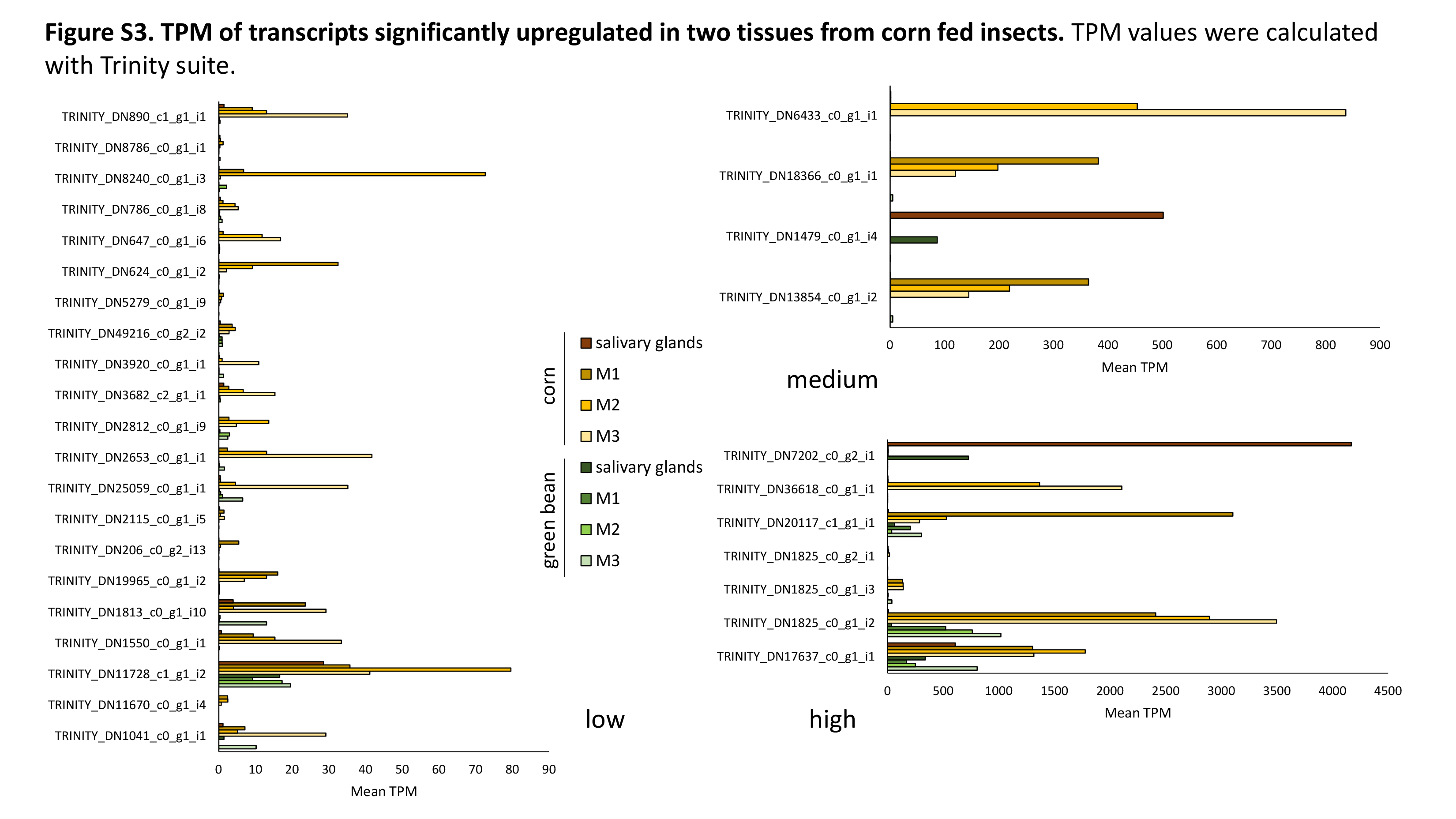

Supplement: Supplementary file 4 [file Image_3.TIF]
