# Supplementary material for: Transcription and Activity of Digestive Enzymes of Nezara viridula Maintained on Different Plant Diets
Source: Front Physiol. 2020 Jan 8;10:1553. doi: 10.3389/fphys.2019.01553 (PMC6960134; doi:10.3389/fphys.2019.01553)
Supplement: Supplementary file 5 [file Table_1.DOCX]

Supplementary Material

## Supplementary Table

**Table S1.** Primers used for amplification of GFP fragment from pGLO for in vitro transcription.

| **Primer** | **Sequence** |
| --- | --- |
| pGLO-GFP Fwd | TAATACGACTCACTATAGGGAG AGGTGATGCTACATACGGAAAG |
| pGLO-GFP Rev | TAATACGACTCACTATAGGGAG ACAGGTAATGGTTGTCTGGTAAA |
